# Tumor lysis syndrome signal with the combination of encorafenib and binimetinib for malignant melanoma: a pharmacovigilance study using data from the FAERS database

**DOI:** 10.3389/fphar.2024.1413154

**Published:** 2024-09-09

**Authors:** Shuang Xia, Jing-Wen Xu, Kang-Xin Yan, Yoshihiro Noguchi, Mayur Sarangdhar, Miao Yan

**Affiliations:** ^1^ Department of Pharmacy, The Second Xiangya Hospital, Central South University, Changsha, China; ^2^ International Research Center for Precision Medicine, Transformative Technology and Software Services, Changsha, China; ^3^ Toxicology Counseling Center of Hunan Province, Changsha, China; ^4^ Department of Pharmacy, Xuzhou Medical University, Xuzhou, China; ^5^ Yali High School International Department, Changsha, China; ^6^ Laboratory of Clinical Pharmacy, Gifu Pharmaceutical University, Gifu, Japan; ^7^ Division of Biomedical Informatics, Cincinnati Children’s Hospital Medical Center, Cincinnati, OH, United States; ^8^ Division of Oncology, Cincinnati Children’s Hospital Medical Center, Cincinnati, OH, United States; ^9^ Department of Pediatrics, University of Cincinnati College of Medicine, Cincinnati, OH, United States

**Keywords:** encorafenib, binimetinib, tumor lysis syndrome, malignant melanoma, pharmacovigilance, FAERS, disproportionality analysis

## Abstract

**Objective:**

To investigate the potential association between tumor lysis syndrome (TLS) and drugs for the treatment of malignant melanoma (MM).

**Methods:**

Reports of TLS recorded in the FDA Adverse Event Reporting System (FAERS) (January 2004–2023q3) were identified. Demographic and clinical characteristics were described, and disproportionality signals were assessed through the Reporting Odds Ratio (ROR) and Information Component (IC). The latency of TLS with anticancer drugs was described based on parametric models. Subgroup analysis was conducted to explore the differences of TLS signals in different age and sex.

**Results:**

We found 5 (1.49%), 59 (17.61%), 79 (23.58%), 19 (5.67%), 13 (3.88%), 13 (3.88%), 33 (9.85%), 49 (14.63%), 16 (4.78%) TLS reports with pembrolizumab, nivolumab, ipilimumab, dabrafenib, vemurafenib, dacarbazine, “encorafenib and binimetinib”, “nivolumab and ipilimumab”, “dabrafenib and trametinib”, respectively. The combination of encorafenib and binimetinib showed the strongest signal of TLS (IC_025_ = 3.98). The median days of latency of TLS with combination of encorafenib and binimetinib is 2 days, which was much shorter than nivolumab (22.0 days) and ipilimumab (21.5 days). TLS cases associated with drugs for MM were predominantly recorded in females and aged 25–65 years. After excluding confounding factors such as pre-existing diseases and co-treated drugs, the disproportionate signal of TLS with “encorafenib and binimetinib” remained strong.

**Conclusions:**

Stronger disproportionate signal of TLS was detected in MM patients using the combination of encorafenib and binimetinib than other drugs. Further research is needed to investigate the underlying mechanisms and identify patient-related predisposing factors to support safe prescribing of the combination of encorafenib and binimetinib.

## 1 Introduction

Malignant melanoma (MM) is a highly malignant tumor that originates from melanocytes in the skin (Guy et al., 2015). Its incidence is on the rise worldwide, making it one of the main types of skin cancer. Representing 1.7% of all cancer diagnoses, melanoma is ranked as one of the most common cancers worldwide, probably reaching 57,000 deaths in the same period (Lopes et al., 2022). The cause of malignant melanoma is not fully understood, but long-term ultraviolet exposure, genetic factors, and immune system abnormalities are known risk factors. At present, the treatment options for malignant melanoma include traditional therapies (dacarbazine, high-dose interleukin-2), immune checkpoint inhibitors (single-agent nivolumab, ipilimumab, and combination of nivolumab plus ipilimumab), targeted therapies (single-agent vemurafenib and dabrafenib, and combination of combination of encorafenib plus binimetinib; dabrafenib plus trametinib; cobimetinib plus vemurafenib) as well as one intralesional modified oncolytic herpes virus talimogene laherparepvec (Swetter et al., 2021). Immune checkpoint blockade strategies targeting the PD-1 and CTLA-4 co-inhibitory receptors, and MAP kinase (MAPK) molecular targeted therapy directed at oncogenic BRAF and MEK signaling pathways. Both approaches have proven effective in the treatment of advanced melanoma (Jenkins and Fisher, 2021).

Tumor lysis syndrome (TLS) is a rare and potentially fatal disease that is often associated with anti-cancer therapy (McBride and Westervelt, 2012). The pathogenesis of TLS includes two aspects: on the one hand, tumor cells produce a large number of metabolites such as uric acid and potassium ions during the rapid breakdown process, and on the other hand, the patient’s excretory organs such as kidneys and liver are not functional enough to quickly remove these metabolites, resulting in their accumulation in the body (Durani and Hogan, 2020). Typical features of TLS include hyperuricemia, hyperkalemia, hypocalcemia, hyperphosphatemia, etc. These metabolic disorders can lead to serious complications (Howard et al., 2016) such as renal insufficiency, arrhythmias, and even life-threatening acute kidney injury. Previous review showed that MM patients had a low incidence of TLS (Kelkar and Wang, 2021).

Studies have found that the combination of encorafenib and binimetinib, a treatment option that can effectively treat malignant melanoma, may cause TLS. Although two studies (Tachibana et al., 2021; Byron et al., 2020) have reported that co-administration of encorafenib and binimetinib may cause TLS, the sample size was small, and these observations need to be confirmed in larger studies. Large pharmacovigilance databases, such as the FAERS and WHO Vigibase, could provide a broader perspective to identify signals of potential associations between drugs and adverse events (AEs) by collecting unpublished reports that occur in unselected subjects in the real-world clinical settings.

In this study, the association between co-treatment of encorafenib plus binimetinib and tumor lysis syndrome was investigated using data from the FAERS database.

## 2 Methods

### 2.1 Study design and data sources

This is a retrospective pharmacovigilance study using curated FAERS data from the AERS*Mine* (Sarangdhar et al., 2016) website. AERS*Mine* is a multi-cohort analyzing application designed to mine curated data across millions of patient reports (currently 20, 346, 289) from the FAERS. Several high-impact pharmacovigilance research (Xia et al., 2023; Sarangdhar et al., 2021) utilized data from the AERS*Mine*. The data used for this study was from the first quarter of 2004 to the third quarter of 2023.

In this study, drugs of interest come from FDA-approved drugs for MM, including nivolumab, ipilimumab, trametinib, dabrafenib, vemurafenib, encorafenib, binimetinib, dacarbazine, vemurafenib, pembrolizumab and combinational therapies. The adverse event of interest was tumor lysis syndrome. Ethical approval was not required because this study was conducted by using deidentified data.

### 2.2 Disproportionality analysis

Case/non-case approach was used to calculate the disproportionate signals of TLS with anti-cancer drugs (Faillie, 2019) There are two methods to calculate the disproportionality signal, which is, namely, frequentist and Bayesian statistical approaches. In this study, the disproportionate signals of TLS with regimens for MM are assessed by calculating Reporting Odd Ratios (ROR) and Information Components (IC) (Bate et al., 1998).

The detection criterion is that there is a statistically significant disproportionate signal when the lower limit of the 95% confidence interval (CI) of the ROR (ROR_025_) (Moore et al., 2005; van Puijenbroek et al., 2002) > 1 and the lower limit of the 95% confidence interval of the IC_025_ (IC_025_) were >0.

### 2.3 Descriptive analysis

The clinical features and demographics (report year, reporter, role code, age, gender and outcome) of TLS with anticancer drugs for MM were collected and analyzed.

### 2.4 Sensitivity analysis

In order to exclude the influence of confounding factors on the results of the study and to test the robustness of the disproportionate signals, we performed series of sensitivity analyses. Firstly, when the adverse effects studied are also reported with one or more drugs other than the target drug (the drug of interest), bias due to drug-to-drug competition may occur. By reviewing the literature (Barbar and Jaffer Sathick, 2021; Williams and Killeen, 2019; Wang et al., 2021), we removed TLS cases reported with other drugs (but not anticancer drugs for MM in this study), which help us to reduce competition biases. Secondly, to avoid exposure bias, we limited the reports of drugs as suspicious, i.e., primary suspect and secondary suspect. At the same time, the scope of reporting was limited to reports by health professionals. Thirdly, we excluded some pre-existing diseases, such as renal dysfunction, hyperuricemia, etc., to reduce indication bias. Finally, we calculated the disproportionality signals of TLS using the available Standardized MedDRA Query, broad search, (including 39 Preferred Terms) to better reflect co-reported adverse events.

### 2.5 Subgroup analysis

In the detection of adverse drug reaction signals, subgroup analysis can help to identify potential risk groups, further highlighting those drug-adverse reaction pairs that are overreported in specific subgroups, and thus identifying potential risk groups (Sandberg et al., 2020). A recent review paper (Noguchi and Yoshimura, 2024) summarized detection algorithms for simple two-group comparisons using spontaneous reporting systems, including frequentist statistical approach (relative ROR), Bayesian statistical approach (ICΔ) and Odds Ratio-based method.

In order to explore the treatment effect or prognosis of patients with different characteristics or subgroup criteria, we divided the cases into age and gender, and divided the cases into older than 65 years and less than or equal to 65 years, male and female, and independently explored the effects of age and gender on the TLS signals. In the study, we used the ICΔ as well as its 95% confidence interval (IC_025_ and IC_975)_ to measure the disproportionate signals between subgroups. A significant signal was detected in subgroups if the 95% confidence interval do not include zero. More details about the formula and algorithms could be found in Supplementary File S1. For raw data on subgroup analyses, please refer to Supplementary File S2.

### 2.6 Time-to-onset (TTO) analysis

TTO modeling (Zhang et al., 2017) is the use of parameter distributions to model the time to onset (Nakamura et al., 2015) of adverse reactions of interest (ADRs) with drugs of interest.

We refer to the dataset that has been cleaned from the AERS*Mine* website. The data included reports from 2004 to 2021 q3. The appropriate model (such as Weibull, log-normal, gamma, exponential, etc.) was selected for data analysis, and the most suitable model was determined by the goodness-of-fit test (Maignen et al., 2010). At the same time, duplicates and reports with missing information were removed to obtain more accurate results. More details about the formula and algorithms could be found in Supplementary File S3. For raw data on Time-to-onset analyses, please refer to Supplementary File S4.

### 2.7 Global assessment of the evidence

Causality was assessed using the adjusted Bradford Hill criteria used in epidemiology to assess the causality of the entire evidence (Andreae et al., 2016), including multiple dimensions such as biological plausibility, strength, consistency, specificity, coherence, and analogy (Muganurmath et al., 2018).

With these approaches, we hope to assess evidence for the potential association between drugs for MM and tumor lysis syndrome.

## 3 Result

### 3.1 Disproportionate signals of TLS with drugs for MM

**FIGURE 1:**
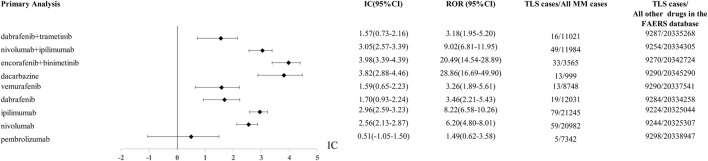
The comparison of Tumor lysis syndrome signal between “encofenib and binitinib” and controls (other anticancer drugs) in FAERS database. ROR, reporting odds ratio; IC, information component; 95%CI, 95% confidence interval; N, number; AEs, adverse events.

Using the AERSMine platform, 9303 TLS cases were detected from the FAERS database from 2004q1 to 2023q3. There are 5, 59, 79, 19, 13, 13, 33, 49, 16 TLS reports with pembrolizumab, nivolumab, ipilimumab, dabrafenib, vemurafenib, dacarbazine, “encorafenib and binimetinib”, “nivolumab and ipilimumab”, “dabrafenib and trametinib”, respectively. The ROR and IC of the above drugs were shown in Figure 1.

**FIGURE 2:**
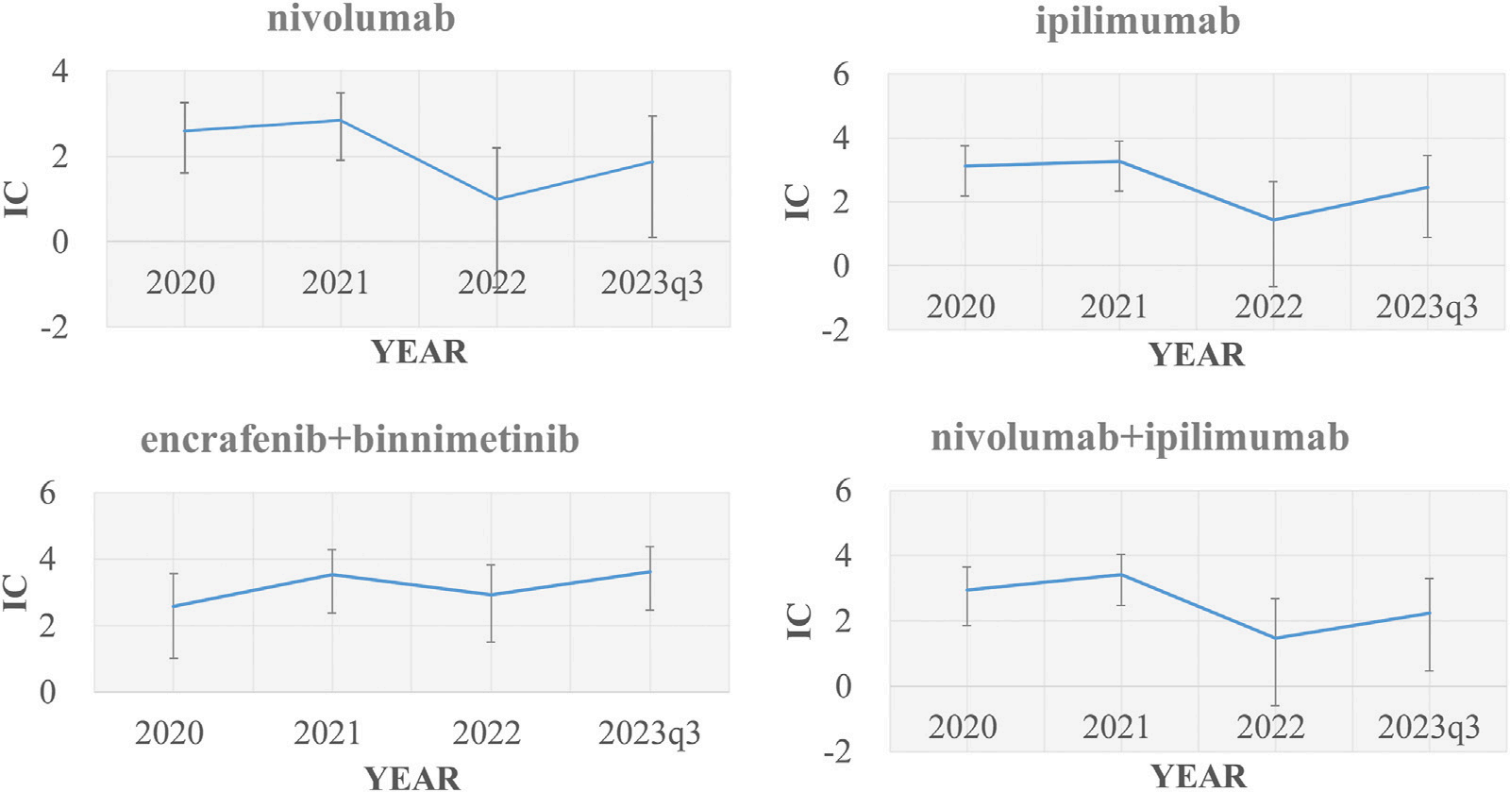
Signal values of nivolumab, ipilimumab, “encorafenib and binimetinib” and “nivolumab and ipilimumab” regarding changes in TLS cases over time (2020, 2021, 2022, 2023q1-2023q3) IC, information component.

Using other drugs in the FAERS database as the comparator, we found the combination of encorafenib and binimetinib showed the most significant disproportionate signal of TLS compared to other regimens. The IC value of TLS with nivolumab, ipilimumab, encorafenib, binimetinib in different years was presented in Figure 2.

### 3.2 Clinical characteristics of TLS cases with regimens for MM

TLS cases of “encorafenib and binimetinib” for melanoma were reported from 2006 to 2023q3 with a total of 33 cases. 87.9% (29/33) of the reported cases were concentrated between 2019 and 2023q3, and 63.6% (21/33) of the cases were reported by health professionals. Deaths were recorded in three cases (9.1%). Life-threatening outcomes were recorded in 15.2% and hospitalizations in 60.6%. 63.6% of TLS with “encorafenib and binimetinib” occurred in 25-65-year-olds and 45.5% in female. The clinical features of TLS with various drugs for MM were shown in Table 1.

**TABLE 1:** Patient characteristics of TLS reports with “encorafenib and binimetinib” and other drugs in the FAERS database.

Categories	Pembrolizumab	Nivolumab	Ipilimumab	Dabrafenib	Vemurafenib	Dacarbazine	Encorafenib + Binimetinib	Nivolumab + Ipilimumab	Dabrafenib + Trametinib
Reports of TLS	5	59	79	19	13	13	33	49	16
Report Year									
2006–2009	0 (0.0%)	0 (0.0%)	6 (7.6%)	0 (0.0%)	11 (84.6%)	7 (53.8%)	0 (0.0%)	0 (0.0%)	0 (0.0%)
2010–2013	1 (20.0%)	7 (11.9%)	12 (15.2%)	5 (26.3%)	1 (7.7%)	0 (0.0%)	0 (0.0%)	4 (8.2%)	2 (12.5%)
2014–2018	4 (80.0%)	20 (33.9%)	27 (34.2%)	2 (10.5%)	1 (7.7%)	0 (0.0%)	4 (12.1%)	15 (30.6%)	2 (12.5%)
2019–2023q3	0 (0.0%)	32 (54.2%)	34 (43.0%)	12 (63.2%)	0 (0.0%)	6 (46.2%)	29 (87.9%)	30 (61.2%)	12 (75.0%)
Reporter									
Healthcare professionals	5 (100.0%)	39 (66.1%)	56 (70.9%)	15 (78.9%)	10 (76.9%)	11 (84.6%)	21 (63.6%)	34 (69.4%)	12 (75.0%)
Other	0 (0.0%)	20 (33.9%)	23 (29.1%)	4 (21.1%)	3 (23.1%)	2 (15.4%)	12 (36.4%)	15 (30.6%)	4 (25.0%)
Age Category									
0–14	0 (0.0%)	0 (0.0%)	0 (0.0%)	0 (0.0%)	0 (0.0%)	0 (0.0%)	0 (0.0%)	0 (0.0%)	0 (0.0%)
15–24	0 (0.0%)	0 (0.0%)	0 (0.0%)	0 (0.0%)	1 (7.7%)	0 (0.0%)	0 (0.0%)	0 (0.0%)	0 (0.0%)
25–65	5 (100.0%)	33 (55.9%)	43 (54.4%)	8 (42.1%)	10 (76.9%)	10 (76.9%)	21 (63.6%)	29 (59.2%)	6 (37.5%)
>65	0 (0.0%)	24 (40.7%)	34 (43.0%)	6 (31.6%)	1 (7.7%)	3 (23.1%)	5 (15.2%)	19 (38.8%)	6 (37.5%)
Data unavailable	0 (0.0%)	2 (3.4%)	2 (2.5%)	5 (26.3%)	1 (7.7%)	0 (0.0%)	7 (21.2%)	1 (2.0%)	4 (25.0%)
Gender									
Male	5 (100.0%)	38 (64.4%)	49 (62.0%)	12 (63.2%)	3 (23.1%)	5 (38.5%)	8 (24.2%)	29 (59.2%)	11 (68.8%)
Female	0 (0.0%)	19 (32.2%)	28 (35.4%)	7 (36.8%)	9 (69.2%)	8 (61.5)	15 (45.5%)	19 (38.8%)	5 (31.3%)
Data Unavailable	0 (0.0%)	2 (3.4%)	2 (2.5%)	0 (0.0%)	1 (7.7%)	0 (0.0%)	7 (21.2%)	1 (2.0%)	0 (0.0%)
Outcome									
Death	2 (40.0%)	16 (27.1%)	31 (39.2%)	10 (52.6%)	2 (15.4%)	3 (23.1%)	3 (9.1%)	8 (16.3%)	9 (56.3%)
Disability	0 (0.0%)	3 (5.1%)	3 (3.8%)	0 (0.0%)	0 (0.0%)	0 (0.0%)	0 (0.0%)	3 (6.1%)	0 (0.0%)
Hospitalization - Initial or Prolonged	4 (80.0%)	49 (83.1%)	62 (78.5%)	16 (84.2%)	11 (84.6%)	5 (38.5%)	20 (60.6%)	41 (83.7%)	14 (87.5%)
Life-Threatening	2 (40.0%)	16 (27.1%)	17 (21.5%)	1 (5.3%)	8 (61.5%)	0 (0.0%)	5 (15.2%)	14 (28.6%)	1 (6.3%)
Other Serious (Important Medical Event)	5 (100.0%)	58 (98.3%)	72 (91.1%)	14 (73.7%)	1 (7.7%)	12 (92.3%)	30 (90.9%)	48 (98.0%)	12 (75.0%)

### 3.3 Sensitivity analysis of TLS signals

To test the robustness in the results, we performed sensitivity analyses. The association between TLS and some regimens (“dabrafenib plus trametinib”, “nivolumab plus ipilimumab”, “encorafenib plus binimetinib”, dacarbazine, vemurafenib, dabrafenib, ipilimumab, nivolumab, pembrolizumab) remained significant even after taking into account possible confounders (competitive bias due to drug interactions, information bias due to reporting health expertise, and information bias due to indications). The detailed disproportionate signals across multiple sensitivity analysis were displayed in Table 2.

**TABLE 2:** Sensitivity analysis of TLS associated with drug of interest (“encorafenib and binimetinib”) and with all other drugs in the FAERS database.

	Corrected for drugrelated competition bias, N0/N1, IC (95% CI)	Corrected for suspect drugs and reports from healthcare professionals, N0/N1, IC (95% CI)	Corrected for TLS (SMQ), N0/N1, IC (95% CI)	Corrected for preexisting disease, N0/N1, IC (95% CI)	Signal consistency/robustness
pembrolizumab	4/5,439 0.59 (−1.17–1.67)	1/4,206 −0.69 (−4.48–1.00)	264/4,206 6.77 (6.57–6.92)	1/4,199 −0.69 (−4.47–1.00)	Weak (1/4)
nivolumab	47/15,040 2.69 (2.20–3.03)	39/13,199 2.60 (2.06–2.98)	946/13,199 7.18 (7.07–7.26)	39/13,165 2.60 (2.07–2.98)	Strong (4/4)
ipilimumab	58/15,322 2.96 (2.53–3.28)	55/13,122 3.09 (2.65–3.41)	948/13,122 7.19 (7.08–7.27)	55/13,110 3.10 (2.65–3.42)	Strong (4/4)
dabrafenib	10/9,427 1.13 (0.05–1.85)	15/7,036 2.06 (1.19–2.66)	544/7,036 7.19 (7.05–7.30)	15/7,012 2.06 (1.19–2.67)	Strong (4/4)
vemurafenib	3/5,547 0.21 (−1.86–1.41)	10/6,678 1.56 (0.49–2.29)	667/6,678 7.55 (7.43–7.65)	10/6,642 1.57 (0.49–2.30)	Intermediate (3/4)
dacarbazine	5/495 2.92 (1.36–3.91)	10/592 3.77 (2.69–4.49)	53/592 6.12 (5.66–6.44)	10/592 3.77 (2.69–4.49)	Strong (4/4)
encorafenib + binimetinib	24/2,413 3.93 (3.25–4.41)	12/578 4.03 (3.05–4.70)	117/578 7.26 (6.96–7.49)	12/566 4.04 (30.6–4.71)	Strong (4/4)
nivolumab + ipilimumab	37/8,569 3.09 (2.54–3.48)	9/2077 2.71 (1.57–3.47)	224/2077 7.27 (7.05–7.43)	9/2073 2.73 (1.58–3.48)	Strong (4/4)
dabrafenib + trametinib	7/8,610 0.76 (−0.54–1.61)	9/2,370 2.58 (1.45–3.25)	297/2,370 7.55 (7.36–7.69)	9/2,350 2.59 (1.45–3.25)	Intermediate (3/4)

### 3.4 Subgroup analysis

**FIGURE 3:**
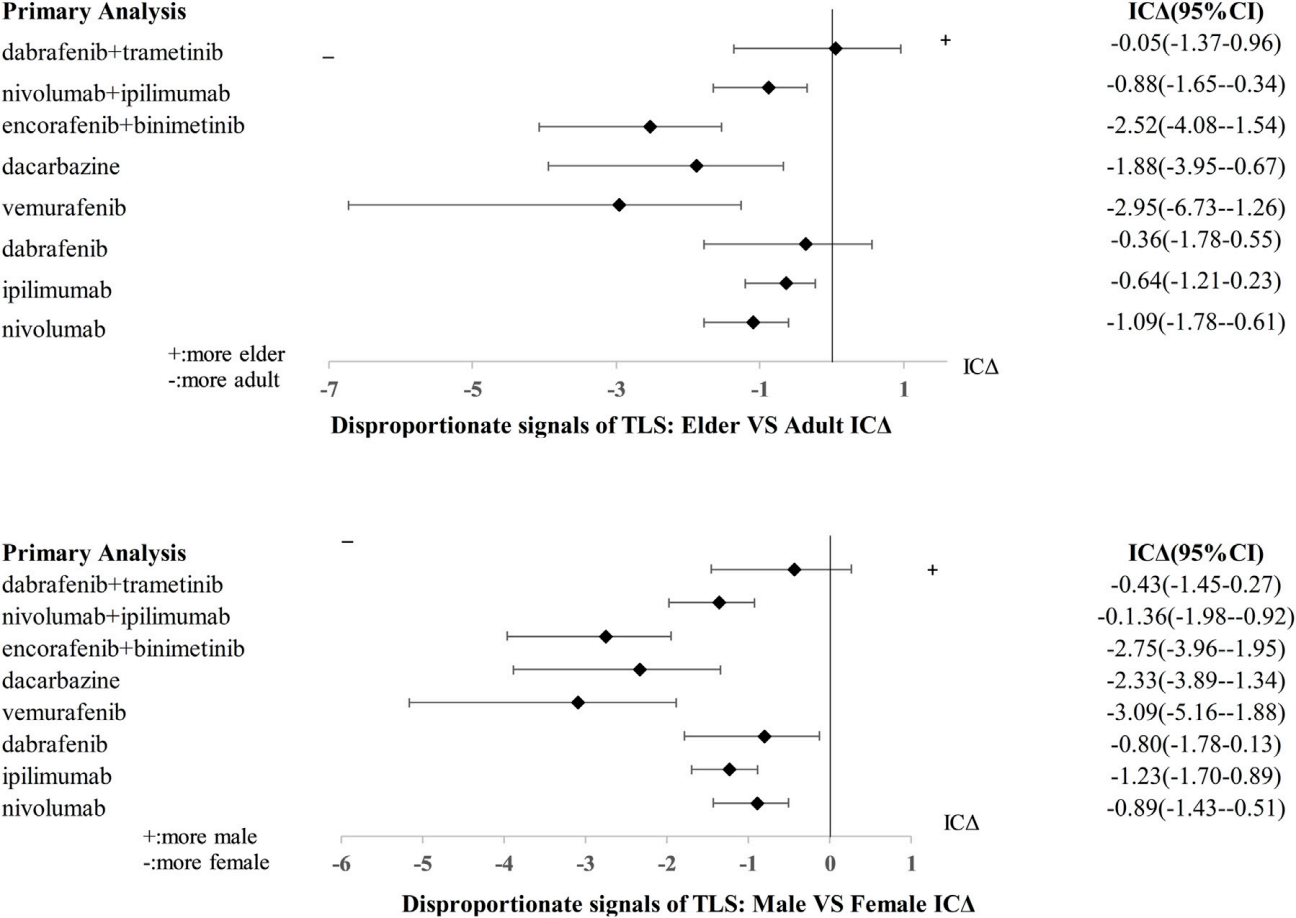
Subgroup analyses (age and sex) of TLS signals with different anti-cancer drugs.

We found that in the case of a combination of encorafenib and binimetinib, women (ICΔ_975_ = −1.95) were more likely to have TLS adverse events than men, patients aged 25–65 years old (ICΔ_975_ = −1.52) were more likely to have TLS events. Detailed subgroup (age and gender) analysis of TLS reports with drugs for MM was shown in Figure 3.

### 3.5 Time-to-onset (analyses)

**FIGURE 4:**
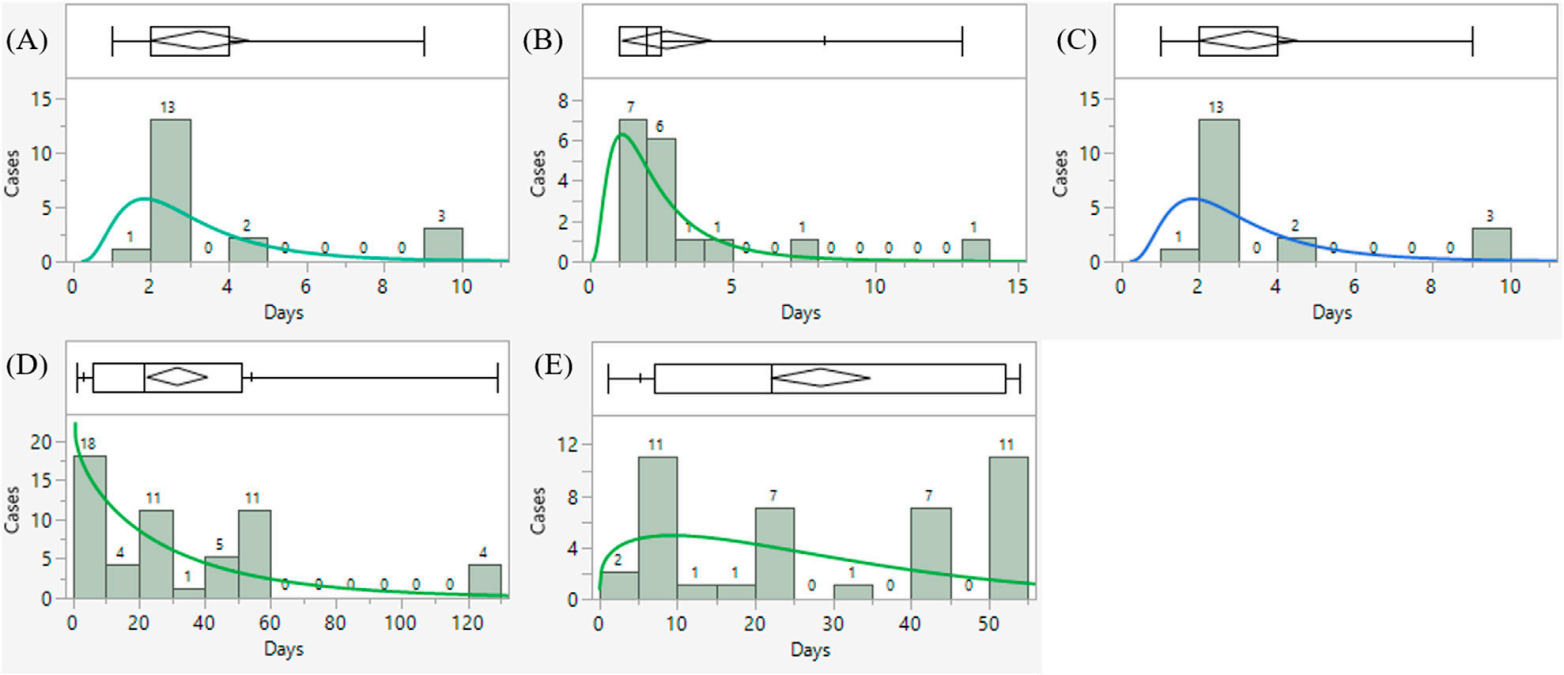
Time to onset analysis of tumor lysis syndrome associated with **(A)** binimetinib **(B)** dabrafenib **(C)** encorafenib **(D)** ipilimumab **(E)** nivolumab in the FAERS database (choose the best model after goodness-fit distribution test).

From our results, the median time-to-onset of TLS with nivolumab, ipilimumab, encorafenib, binimetinib, dabrafenib was 22.0, 21.5, 2.0, 2.0, 2.0 days, respectively (Table3). A detailed analysis of the relevant drugs is provided in Figure 4.

**TABLE 3:** Time-to-onset analysis of TLS associated with anti-cancer drugs for MM in the FAERS database.

Categories	Binimetinib	Dabrafenib	Encorafenib	Ipilimumab	Nivolumab
Reports of TLS	19	18	19	54	41
Median days	2.0	2.0	2.0	21.5	22.0
Scale parameter α (95% CI)	1.10 (0.19–2.01)	0.97 (0.68–1.25)	0.97 (0.68–1.25)	30.79 (22.50–41.62)	30.60 (23.48–39.37)
Shape parameter β (95% CI)	0.85 (0.51–1.85)	0.60 (0.45–0.85)	0.60 (0.45–0.85)	0.93 (0.75–1.14)	1.28 (0.98–1.64)

α, scale parameter, represents the scale of the distribution function as the quantile in which 63.2% of AEs, occur. β, shape parameter, could be used to confirm.

the distribution type: early failure type (β < 1), random failure type (95% CI, of β include 1), and wear-out type (β > 1).

95% CI, 95% confidence interval; AEs, adverse events; FAERS, US, food and drug administration adverse event reporting system; TLS, tumor lysis syndrome.

### 3.6 Global assessment of the evidence

By evaluating adopted Bradford Hill criteria, including association strength, consistency, specificity, temporal relationships, experimental evidence, coherence and analogy, we found the associations between “encorafenib plus binimetinib” and tumor lysis syndrome met the causality assessment assessed by the Bradford Hill criteria. More details were shown in Table 4.

**TABLE 4:** Global assessment through adapted Bradford Hill Criteria.

Criteria	Description	Source/method
Strength of the association	Although IC is not a measure of risk, it shows a strong disproportionate signal in the disproportionate signal analysis	Disproportionate reporting of TLS with “encorafenib and binimetinib” in the FAERS database
Analogy	Other anti-cancer drugs (such as venetoclax) have also demonstrated this association	Literature
Biological plausibility/empirical evidence	Not applicable	Not applicable
Consistency	Published two case reports studies support the potential association of TLS with “encorafenib and binimetinib”	Literature
Exclusion of biases/confounders	The statistical disproportionality persisted and was strong after sensitivity analysis, excluding the influence of confounders	Disproportionality
Specificity	This study did not detect significant TLS signals with other two BRAF/MEK combinations (dabrafenib plus trametinib, or cobimetinib plus vemurafenib). A drug-specific effect (rather than a class-effect) was considered	Disproportionality
Temporal relationship	All TLS events with “encorafenib and binimetinib” manifested after the suspected drug was administered in both the pharmacovigilance analysis and published case reports	Time-to-onset analysis and literature
Reversibility	This criterion is of limited value here as there is no data on discontinuation and dechallenge in the FAERS database	Not applicable
Coherence	The reasoning about cause and effect as present in the aforementioned criteria	Literature

FAERS, US, food and drug administration adverse event reporting system; IC, information components; TLS, tumor lysis syndrome.

These items were not included in the original Bradford Hill Criteria.

## 4 Discussion

This is the first pharmacovigilance study to investigate the disproportionate signals of TLS with the combination of encorafenib and binimetinib for the treatment of MM. We have identified three new key findings that provided additional information to the safe administration of the combination of encorafenib and binitenib in the treatment of malignant melanoma.

First, by exploring the FAERS database and performing a disproportionality analysis, we found that the disproportionate signals between TLS and two combinational therapies (“encorafenib and binimetinib”, “nivolumab and ipilimumab”) were strong, and much higher than other drugs in the FAERS database, including other anticancer drugs for the treatment of malignant melanoma. We verified the robustness of the signals through four sensitivity analyses to exclude confounding factors such as drug competition, exposure bias, and information bias. Our data suggested that the combination of encorafenib and binimetinib may significantly increase the signal of TLS compared to other anticancer drugs (e.g., chemotherapy or targeted therapy). Of note, 33 TLS cases with the combination of encorafenib and binimetinib were reported from 2019 to 2023, which showed a rapidly increasing reporting of TLS with this combination therapy. Previous research (Wang et al., 2021) already reported that the combination of nivolumab plus ipilimumab had higher TLS signals compared to monotherapy, which was consistent with our study. In the time-scan analysis (Figure 2), TLS signals with nivolumab, ipilimumab, nivolumab plus ipilimumab became insignificant in 2022. But TLS signals with the combination of encorafenib and binimetinib kept robust from 2019 to 2023. And the following sentences were added into the end of second paragraph of the discussion: “Considering that TLS have some typical symptoms, such as raised creatinine, hyperkalemia, hypocalcemia, hyperphosphatemia, hyperuricemia and renal impairment. We compared the disproportionate signals of all preferred terms within the Standardised MedDRA Queries (SMQ, broad) associated with different regimens for the treatment of MM. We found that the combination of encorafenib and binimetinib had higher disproportionate signals of blood creatinine increased, hyperkalaemia and renal impairment compared to other regimens (Supplementary File S5). This is consistent with the disproportionate signal of TLS.

Second, we found that the combination of “encorafenib and binimetinib” had a shorter onset time than “nivolumab and ipilimumab” (median days, 2.0 days vs. 22.0 days). By searching the website of Drugbank, we found that the mean terminal half-life (t1/2) of binimetinib, encorafenib, nivolumab and is 3.5 h (28.5%), 3.5 h (17%), 20 days and 14.7 days, respectively. This may partly explain the difference of TLS latency between “encorafenib and binimetinib” and “nivolumab and ipilimumab”.

Both “encorafenib and binimetinib” and “nivolumab and ipilimumab” have strong disproportionate signals of TLS, the shorter onset time of TLS with “encorafenib and binimetinib” is of great concern to patients and clinicians. Because encorafenib and binimetinib are orally administered while nivolumab and ipilimumab are intravenous injection. It would be more difficult to manage if TLS occur when patients orally administer the combination of encorafenib and binimetinib at home.

Third, through subgroup analysis, we found that people aged 25–65 years who were treated with “encolafenib and binimetinib” were more likely to have TLS compared with other age groups. Previous research (Guy et al., 2015) showed that the peak incidence of MM is around the age of 50. This may partly explain the difference of TLS signals in different ages. This study also identified that females were more likely to develop TLS with the co-treatement of encolafenib and binimetinib. Previous literature (Ji et al., 2013; Blum et al., 2011) showed that female gender was the most influential of all risk factors identified for TLS occurrence after flavopiridol treatment with an OR of 8.6 (95% CI: 2.6–27.7) because females displaying higher flavo-G exposure than males. According to the FDA approved drug labels, encorafenib is primarily metabolized by CYP3A4 (83%) and to a lesser extent by CYP2C19 (16%) and CYP2D6 (1%). The primary metabolic pathway is glucuronidation with UGT1A1 contributing up to 61% of the binimetinib metabolism. Males and females may have different metabolism capacity on encorafenib and binimetinib, which may affect the clearance of encorafenib and binimetinib in the body. This may partly explain the higher disproportionate signals of TLS in females. Further studies are needed to investigate the influence of age and gender on the occurrence of TLS associated with the combination of encorafenib and binimetinib. Further research is warranted to verify our findings.

This study has some limitations (Noguchi et al., 2021). Firstly, the reports in the FAERS database are heterogeneous (from both healthcare professionals and non-health care professionals), which may affect the quality of data. Secondly, reports from the FAERS could not provide more clinical information (for example, the stages of MM), which may affect the disproportionate signals. Thirdly, there is under-reporting bias, channel bias in the data from FAERS (Faillie, 2019). Therefore, it is suggested that future studies should use more comprehensive data sources, including clinical trials and observational studies, to further validate our findings.

## 5 Conclusions

In this study, we conducted a pharmacovigilance study on tumor lysis syndrome signaling in anticancer drug therapy for MM based on the FAERS database. The study found that among the treatments for MM, the commonly used treatment method—“encorafenib and binimetinib” is prone to tumor lysis syndrome (TLS)-related adverse reactions. Moreover, in focused analysis after excluding confounding factors, we not only corroborated this finding but also identified that the TLS onset time of “encorafenib and binimetinib (2.0 days)” was shorter than that of “nivolumab (22.0 days) and ipilimumab (21.5 days)”. Subgroup analyses revealed that middle-aged patients, particularly women, are more likely to experience TLS when taking the combination of encorafenib and binimetinib. TLS is known to be a serious complication associated with anticancer therapy. This study outlines the TLS profiles for common MM drugs for improved safety in clinical practice. TLS related indicators should be closely monitored in patients receiving anticancer therapy, and timely and effective interventions should be taken to reduce the risk of TLS. In addition, future studies should further explore the pathogenesis and prevention strategies of TLS to improve the quality of life and prognosis of patients.

## Data Availability

All data are publicly available on the website of AERSMine, a curated FAERS database: https://research.cchmc.org/aers/.

## Supplementary material

SUPPLEMENTARY FILE S1 Calculation methods of ROR, IC and IC delta.

SUPPLEMENTARY FILE S2 Raw data of subgroup analysis.

SUPPLEMENTARY FILE S3 Raw data of time to onset analysis.

SUPPLEMENTARY FILE S4 Fit of goodness results for parametric models in time-to-onset analsis.

SUPPLEMENTARY FILE S5 The disproportionate signals of all PT terms within the “tumour lysis syndrome” SMQ (broad) with different drug therapies for the treatment of malignant melanoma.
