# Supplementary material for: Tumor lysis syndrome signal with the combination of encorafenib and binimetinib for malignant melanoma: a pharmacovigilance study using data from the FAERS database
Source: Front Pharmacol. 2024 Sep 9;15:1413154. doi: 10.3389/fphar.2024.1413154 (PMC11417307; doi:10.3389/fphar.2024.1413154)
Supplement: Supplementary file 3 [file Table1.DOCX]

**Title:**

**Tumor lysis syndrome signal with the combination of encorafenib and binimetinib for malignant melanoma: a pharmacovigilance study using data from the FAERS database**

**Part one: Calculation method details in this pharmacovigilance study**

**1. Calculation method of ROR, IC is as following:**

**Table1**:2×2 contingency table for signal detection

|  | **Targeted AEs** | **Other AEs** | **Total** |
| --- | --- | --- | --- |
| **Targeted drugs** | N_11_ | N_10_ | N_1+_ |
| **Other drugs** | N_01_ | N_00_ | N_0+_ |
| **Total** | N_+1_ | N_+0_ | N_++_ |

$$\begin{aligned} ROR=\frac{\left( N_{11}*N_{00} \right)}{\left( N_{01}*N_{10} \right)}\#\left( 1 \right) \end{aligned}$$

$$\begin{aligned} \mathrm{IC}\left( 95\%confidence interval \right)=e^{\ln\left( \mathrm{ROR} \right)\pm1.96\sqrt{\left( \frac{1}{N_{11}}+\frac{1}{N_{10}}+\frac{1}{N_{01}}+\frac{1}{N_{00}} \right)}} \#\left( 2 \right) \end{aligned}$$

The statistical formula is as follows to calculate IC,

$$\begin{aligned} IC=\log_{2}\left( \frac{N_{\mathrm{observed}}+ 0.5}{N_{\mathrm{expected}}+ 0.5} \right)\#\left( 3 \right) \end{aligned}$$

$$\begin{aligned} IC=\frac{\left( N_{\mathrm{drug}}*N_{\mathrm{effect}} \right)}{N_{\mathrm{total}}}\#\left( 4 \right) \end{aligned}$$

$$\begin{aligned} \mathrm{IC}_{025}=\log_{2}\left( \frac{N_{\mathrm{observed}}+ 0.5}{N_{\mathrm{expected}}+ 0.5} \right)-3.3*\left( N_{\mathrm{observed}}+0.5 \right)^{-\frac{1}{2}}-2*\left( N_{\mathrm{observed}}+0.5 \right)^{-\frac{3}{2}}\#\left( 5 \right) \end{aligned}$$

$$\begin{aligned} \mathrm{IC}_{975}=\log_{2}\left( \frac{N_{\mathrm{observed}}+ 0.5}{N_{\mathrm{expected}}+ 0.5} \right)+2.4*\left( N_{\mathrm{observed}}+0.5 \right)^{-\frac{1}{2}}-0.5*\left( N_{\mathrm{observed}}+0.5 \right)^{-\frac{3}{2}}\#\left( 6 \right) \end{aligned}$$

N_expected_: the number of case reports expected for the drug-adverse effect combination.

N_observed_: the actual number of case reports for the drug- adverse effect combination.

N_drug_: the number of case reports for the drug, regardless of adverse effects.

N_effect_: the number of case reports for the adverse effect, regardless of the drug.

N_total_: the total number of case reports in the database.

**2. Calculation method of ICΔ is as following:**

Table 2 The 4 × 2 contingency table for two-group comparisons

|  | Target adverse  event | Other adverse  events | Total |
| --- | --- | --- | --- |
| Group_A_ |  |  |  |
| Target drug | n_A11_ | n_A10_ | n_A1+_ |
| Other drugs | n_A01_ | n_A00_ | n_A0+_ |
| Group_not A_ |  |  |  |
| Target drug | n_not A11_ | n_not A10_ | n_not A1+_ |
| Other drugs | n_not A01_ | n_not A00_ | n_not A0+_ |
| Total | n_+1_ | n_+0_ | n_++_ |

N, the number of reports

$$\begin{aligned} OE=\frac{O(Observed)}{E(Expected)}=\frac{n_{11}}{n_{+1}n_{1+}/n_{++}}\#\left( 7 \right) \end{aligned}$$

$$\begin{aligned} {OE}_{\Delta}=\frac{{OE}_{A}}{{OE}_{notA}}=\frac{O_{A}}{E_{A}O_{notA}/E_{notA}}=\frac{O_{A}}{E^{*}}\#\left( 8 \right) \end{aligned}$$

$$\begin{aligned} \mathrm{IC}_{\Delta}=\log_{2}\left( \frac{O_{A}+ 0.5}{E_{*}+ 0.5} \right)\#\left( 9 \right) \end{aligned}$$

The 95% CrI of ICΔ was calculated using the same IC method.

**Part two: Sensitivity analysis sets**

1. **Exclude drugs know to increase the risk of tumor lysis syndrome, such as nephrotoxic drugs.**

"x-ray contrast media, iodinated" "calcineurin inhibitors" "sulfamethoxazole and trimethoprim" "vancomycin" "antiinflammatory agents, non-steroids" "paracetamol" "acetazolamide" "aciclovir" "allopurinol" "acetylsalicylic acid" "amitriptyline" "aminoglycoside antibacterials" "amphotericin b" "angiotensin ii receptor blockers (arbs), plain" "ace inhibitors, plain" "benzodiazepine related drugs" "beta-lactam antibacterials, penicillins" "carbenicillin" "third-generation cephalosporins" "first-generation cephalosporins" "second-generation cephalosporins" "fourth-generation cephalosporins" "other cephalosporins and penems" "cimetidine" "cisplatin" "clopidogrel" "cocaine" "cortisone" "cyclophosphamide" "ciclosporin" "diphenhydramine" "penicillamine and similar agents" "furosemide" "ganciclovir" "haloperidol" "indinavir" "interferon alfa natural" "lansoprazole" "lithium" "methadone" "metamfetamine" "mitomycin" "naproxen" "omeprazole" "pamidronic acid" "pantoprazole" "penicillamine" "pentamidine isethionate" "phenformin" "phenacetin" "phenytoin" "probenecid" "quinine" "quinolone antibacterials" "rifampicin" "ranitidine" "hmg coa reductase inhibitors" "sulfonamides, plain" "tetracyclines" "low-ceiling diuretics, thiazides" "tolbutamide" "brentuximab vedotin" "obinutuzumab" "rituximab" "ofatumumab" "dasatinib" "ibrutinib" "fludarabine" "ibrutinib" "acalabrutinib" "zanubrutinib" ("ipilimumab" and "nivolumab") "tacrolimus"

1. **exclude some pre-existing diseases may increase the risk of tumor lysis syndrome.**

"renal insufficiency" "chronic renal insufficiency" "dehydration" "tumour lysis syndrome" "hypotension" "obstructive uropathy" "renal impairment" "splenomegaly" "hepatomegaly" "blood lactate dehydrogenase increased" "end stage renal disease" "uremia" "renal failure and impairment" "oliguria" "urinary tract obstruction" "nephropathy" "hyperuricemia" "hyperphosphatemia" "hypovolemia" "white blood cell count increased" "hypocalcemia" "hyperkalemia" "acute kidney injury" "bone marrow transplant" "bone marrow failure" "bone marrow disorder"

**References:**

**For part one in this file:**

1. Rothman KJ, Lanes S, Sacks ST. The reporting odds ratio and its advantages over the proportional reporting ratio. Pharmacoepidemiol Drug Saf. 2004;13(8):519-23.

2. Noren GN, Hopstadius J, Bate A. Shrinkage observed-to-expected ratios for robust and transparent large-scale pattern discovery. Stat Methods Med Res. 2013;22(1):57-69.

3. Noren GN, Sundberg R, Bate A, Edwards IR. A statistical methodology for drug-drug interaction surveillance. Stat Med. 2008;27(16):3057-70.

4. Noguchi Y, Yoshimura T. Detection Algorithms for Simple Two-Group Comparisons Using Spontaneous Reporting Systems. Drug Saf. 2024 Feb 22. doi: 10.1007/s40264-024-01404-w. Epub ahead of print.

**For part two:**

4. Barbar T, Jaffer Sathick I. Tumor Lysis Syndrome. Adv Chronic Kidney Dis. 2021;28(5):438-46 e1.

5. Williams SM, Killeen AA. Tumor Lysis Syndrome. Arch Pathol Lab Med. 2019;143(3):386-93.

6. Wang L, Li X, Zhao B, Mei D, Jiang J, Duan J. Immune Checkpoint Inhibitor-Associated Tumor Lysis Syndrome: A Real-World Pharmacovigilance Study. Front Pharmacol. 2021;12:679207.

7. Nephrotoxic Medications ([Nephrotoxic Medications - StatPearls - NCBI Bookshelf (nih.gov)](https://www.ncbi.nlm.nih.gov/books/NBK553144/)).
