# Supplementary material for: Tumor lysis syndrome signal with the combination of encorafenib and binimetinib for malignant melanoma: a pharmacovigilance study using data from the FAERS database
Source: Front Pharmacol. 2024 Sep 9;15:1413154. doi: 10.3389/fphar.2024.1413154 (PMC11417307; doi:10.3389/fphar.2024.1413154)
Supplement: Supplementary file 4 [file Table4.DOCX]

**Tumor lysis syndrome signal with the combination of encorafenib and binimetinib for malignant melanoma: a pharmacovigilance study using data from the FAERS database**

The performance test of goodness-of-fit among four parametric distribution models, used for reported time-to-onset analysis.

For TLS with binimetinib:

| **Model name** | **AICc** | **BIC** | **-2 Log Likelihood** | **Selection evidence** |
| --- | --- | --- | --- | --- |
| Weibull | 83.38 | 84.52 | 78.63 | Log-normal model presented the smallest and -2 Log Likelihood, showed good fitness |
| Log-normal | 75.90 | 77.04 | **71.15** |  |
| Gamma | 80.75 | 81.89 | 76.00 |  |
| exponent | 85.18 | 85.89 | 82.94 |  |

For TLS with dabrafenib:

| **Model name** | **AICc** | **BIC** | **-2 Log Likelihood** | **Selection evidence** |
| --- | --- | --- | --- | --- |
| Weibull | 72.04 | 72.85 | 67.18 | Log-normal model presented the smallest AICc, BIC and -2 Log Likelihood, showed good fit |
| Log-normal | 64.70 | 65.51 | **59.85** |  |
| Gamma | 70.62 | 71.43 | 65.77 |  |
| exponent | 70.11 | 70.68 | 67.84 |  |

For TLS with encorafenib:

| **Model name** | **AICc** | **BIC** | **-2 Log Likelihood** | **Selection evidence** |
| --- | --- | --- | --- | --- |
| Weibull | 83.38 | 84.52 | 78.63 | Log-normal model presented the smallest -2 Log Likelihood, showed good fit |
| Log-normal | 75.90 | 77.04 | **71.15** |  |
| Gamma | 80.75 | 81.89 | 76.00 |  |
| exponent | 85.18 | 85.89 | 82.94 |  |

For TLS with ipilimumab:

| **Model name** | **AICc** | **BIC** | **-2 Log Likelihood** | **Selection evidence** |
| --- | --- | --- | --- | --- |
| Weibull | 485.46 | 489.20 | **481.23** | Weibull model presented the smallest BIC and -2 Log Likelihood, showed good fit |
| Log-normal | 489.63 | 493.38 | 485.40 |  |
| Gamma | 485.54 | 489.28 | 481.30 |  |
| exponent | 483.75 | 485.67 | 481.67 |  |

For TLS with nivolumab:

| **Model name** | **AICc** | **BIC** | **-2 Log Likelihood** | **Selection evidence** |
| --- | --- | --- | --- | --- |
| Weibull | 357.85 | 360.96 | **353.54** | Weibull model presented the smallest -2 Log Likelihood, showed good fit |
| Log-normal | 368.03 | 371.14 | 363.71 |  |
| Gamma | 359.13 | 362.24 | 354.81 |  |
| exponent | 358.83 | 360.44 | 356.73 |  |

TLS, tumor lysis syndrome. AICc, BIC and -2 Log Likelihood are indicator of goodness of fit in JMP. More details about their meaning please refer to [Likelihood, AICc, and BIC (jmp.com)](https://www.jmp.com/support/help/en/17.0/index.shtml#page/jmp/likelihood-aicc-and-bic.shtml)
